# Brief Research Report: Veterinary Student Perspective on COVID-19 and Veterinary Medicine

**DOI:** 10.3389/fvets.2021.723890

**Published:** 2021-10-14

**Authors:** Candice B. Limper, Ariana L. Hinckley-Boltax, Casey L. Cazer

**Affiliations:** ^1^Department of Microbiology and Immunology, Cornell University College of Veterinary Medicine, Ithaca, NY, United States; ^2^Department of Comparative Pathobiology, Cummings School of Veterinary Medicine at Tufts University, Grafton, MA, United States; ^3^Department of Population Medicine and Diagnostic Sciences, Cornell University College of Veterinary Medicine, Ithaca, NY, United States

**Keywords:** one health, veterinary medicine, COVID-19, veterinary student, content analysis

## Abstract

COVID-19 has had significant effects on the field of veterinary medicine. Adaptation to pandemic-related and post-pandemic challenges requires engagement from all levels of the professional pipeline, including veterinary college students. Insights gained from this group may inform curriculum design, help the veterinary profession innovate, maximize opportunities for positive change, and avoid negative outcomes. The current study aimed to understand the potential impacts of the COVID-19 pandemic on veterinary medicine, as foreseen by second-year veterinary students in an online discussion during a public health course in the spring of 2020. Twenty-one percent of the 113 students agreed to participate in this qualitative research study. We used an inductive coding process and distilled the student responses into descriptive themes to capture diverse perspectives and understand possible post-pandemic pathways for the veterinary profession. Four themes emerged from the student discussion posts, describing how veterinarians might be affected by the COVID-19 pandemic: (1) economic and social impacts, (2) adapting to challenges, (3) collaborations to improve public health, and (4) disparities and diversity. These themes are a starting point for discussion and innovation as veterinarians plan for the post-pandemic world; further investigation will provide additional guidance for veterinary leaders.

## Introduction

The COVID-19 pandemic's immediate effects on veterinary medicine included substantial changes in operating protocols in the face of shortages of drugs, personal protective equipment (PPE), and staff. Veterinarians adopted social distancing measures, changed staff management practices, created new animal handling techniques, and increased their use of telemedicine to prevent the transmission of SARS-CoV-2 (1, 2). In addition, veterinarians found themselves in the spotlight as animal health experts when SARS-CoV-2 positive animals were identified (3). They demonstrated their expertise in diagnostic testing and outbreak response (4, 5). At the very beginning of the pandemic, we engaged second-year veterinary students in a discussion of how the COVID-19 pandemic may impact veterinarians and veterinary medicine. These students were concurrently studying the public health role of veterinarians; thus, this topic was an appropriate and timely addition to their course.

Veterinary student perspectives and opinions can provide guidance for the veterinary profession, often by identifying educational gaps. For example, one study elicited the opinions of final year veterinary students on climate change and found that students wanted to be leaders on environmental sustainability issues related to clinical practice but they lacked opportunities for education and training in this area (6). Educators have also elicited student opinions on the importance of public health, epidemiology, and One Health curricula (7–9). Our objective is to distill student discussions of COVID-19 and veterinary medicine into descriptive themes using content analysis to explore the potential long-term effects of the COVID-19 pandemic on the veterinary profession.

## Methods

### Data Collection

Second-year veterinary students enrolled in a required 1.5 credit public health course (see Supplementary Materials for syllabus) participated in an online discussion of how the COVID-19 outbreak might affect veterinarians; 21% of students *(* n = 24 out of 113) granted permission for their 48 anonymized posts (two per student) to be used in this exploratory study. Twenty out of 24 participants elected to provide demographic data and career plan information. The seven-week course (March 30, 2020–May 15, 2020) was delivered online synchronously through virtual lectures and asynchronously with independent study topics. The course instructor provided an initial discussion prompt and six potential discussion topics; additional topics were later suggested, although the majority of students identified their own topics (Supplementary Materials). This project was reviewed and approved by the Cornell University Institutional Review Board (protocol ID# 2005009621).

### Analysis

Thematic and content analysis is a descriptive process of identifying, analyzing, and reporting patterns in a data set guided by standardized recommendations that reduce the bias associated with drawing conclusions from written or spoken words (10). It is used pervasively in education and other social science literature to better understand a subject through the lens of individuals who experience it and is gaining traction in healthcare fields (11). This qualitative data analysis method is guided by standardized recommendations that reduce the bias associated with drawing conclusions from written or spoken words (10). Approaches to thematic analysis vary, and can be summarized into inductive vs. deductive approaches. In inductive approaches, themes emerge from open-ended inquiry into the data set, while in deductive approaches, pre-identified hypotheses can be explored through inquiry into the data set (10). In veterinary education, this method has been used to explore phenomena encountered by students, residents, faculty, and others in forms such as focus groups, feedback surveys, and assignments (12–15).

We first used an inductive coding process and achieved code saturation (16) by analyzing posts from ten students, randomly selected with Microsoft Excel's random number generator. Each post was assigned to each of two coders (CC and CL), who independently created and assigned codes using ATLAS.ti (version 8, Scientific Software Development GmbH, Berlin, Germany). Each sentence was given one or more codes based on its manifest content (i.e., descriptive written material rather than implied or abstracted meaning). These preliminary codes were discussed, defined, and grouped in a codebook (Supplementary Table 1). Both coders then independently analyzed all posts and independently identified themes that bridged several codes or code categories. Code assignments and themes were compared and discussed by collecting and grouping relevant direct quotations, resulting in three main themes. We then used a deductive coding process following the same procedure as described above to investigate a fourth theme, centered around diversity, equity, and inclusion, in response to the many observed intersections between the pandemic and social justice issues in the spring of 2020. We used the COREQ checklist (17) to document the research process; complete checklist information is provided in the Supplementary Materials.

Code co-occurrence was analyzed by merging the code assignments from the two coders and comparing code groups applied to each sentence. A code co-occurrence heatmap and word cloud were created in RStudio (version 4.0.1) (18). The word cloud was made by removing common English words (Stopwords – ISO list) and merging words with common stems; word frequencies were normalized by dividing the word frequency by the maximum word frequency. Participant demographic data was analyzed descriptively using RStudio.

## Results and Discussion

The average age of the participating second-year veterinary students was 27 with a median of 25, slightly older than the general U.S. veterinary student population (19). Seventy-five percent were female, 85% were white, 15% were Hispanic or Latino, and 40% were first-generation college students. This is slightly less female, more white, and more first-generation college students than the overall U.S. veterinary student class of 2022 (20). Twenty-five percent of the students reported growing up in a rural area, 65% in a suburban area, and 10% in an urban area; the overall class of 2022 was moderately more urban and less suburban (20). The majority (50%) were interested in small animal medicine (including shelter medicine) 35% were interested in zoo, wildlife, or exotic medicine (one student was interested in both small animal and exotic practice), 10% were interested in mixed practice, and 10% were focused on large animals. After graduation, 40% intended to do an internship or residency and 40% intended to join a private practice. This group of students were less likely to be interested in private practice than the general veterinary student population and more likely to be interested in alternative career paths (industry −10%, government−5%, non-profits−5%) (19). This suggests that the students most interested in the COVID-19 discussion were those already thinking outside the box of traditional veterinary roles.

**Figure 1:**
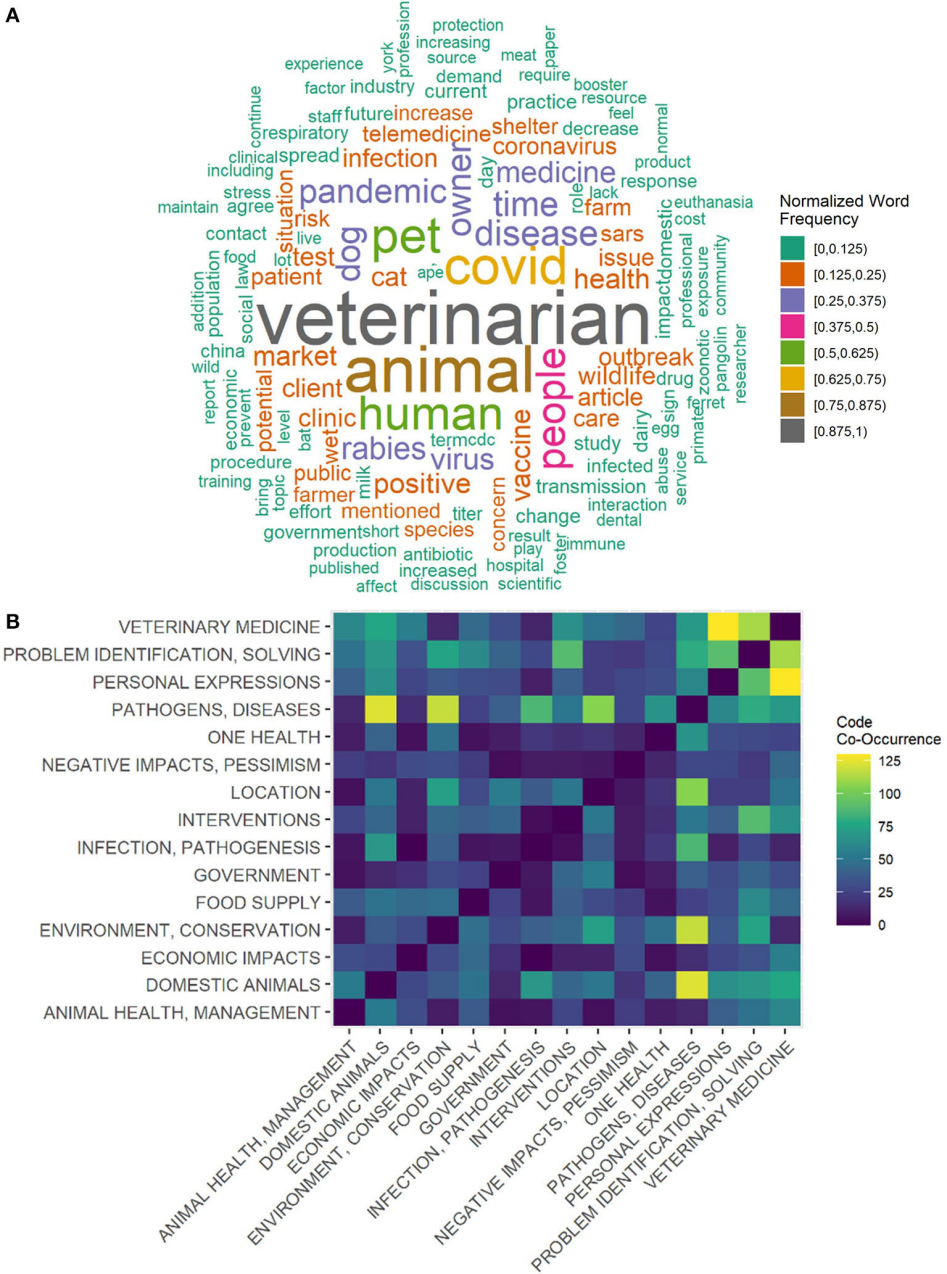
Student discussion post summary. **(A)** Word cloud of the student discussion posts. Word size is proportional to the frequency at which the word occurred in the discussion posts and word color indicates binned normalized frequencies. **(B)** Heatmap of code group co-occurrences. The 15 most commonly used code-groups are shown. Code group definitions are given in Supplementary Table 1.

**Figure 2:**
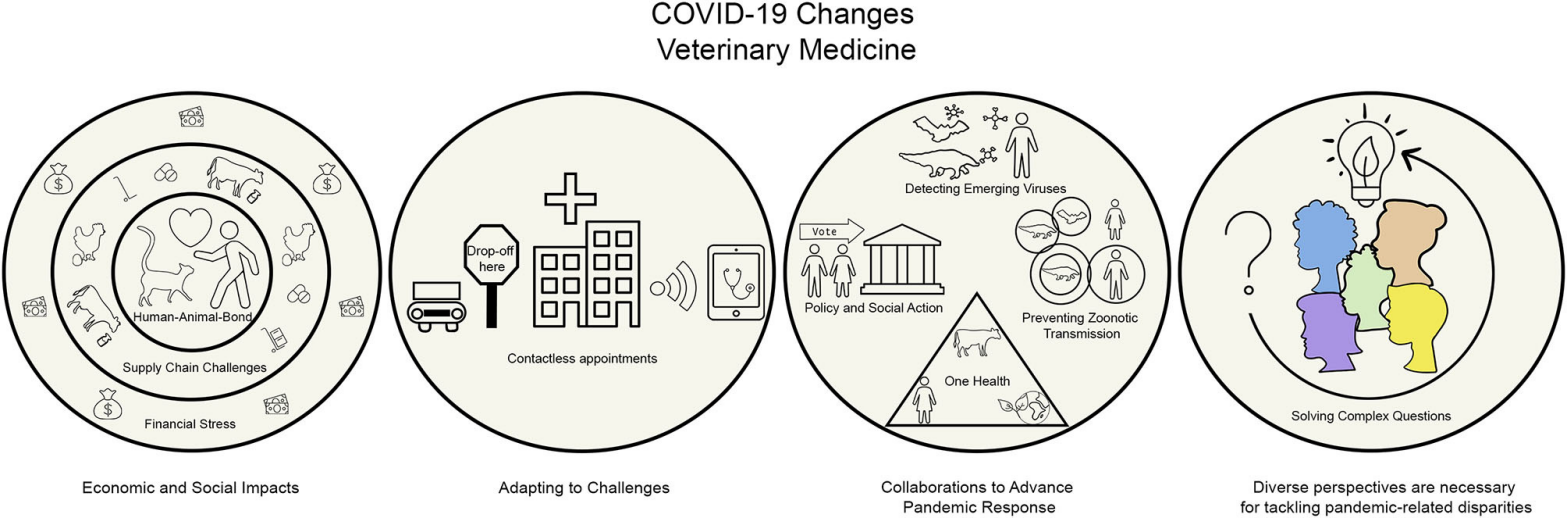
Visual summary of the discussion post content analysis and themes.

The average discussion post was 454 words with three references cited. The words “human” and “people” occurred almost as much as “pet” and “animal” (Figure 1A), reflecting some influence of the course topic and the One Health aspects of the pandemic. Students frequently discussed solving problems in veterinary medicine and their personal experiences as veterinarians-in-training (Figure 1B). The interactions between domestic animals, the environment, and pathogens were also common (Figure 1B). Only seven of the provided discussion prompts (Supplementary Materials) were utilized by the participating students; the majority of the posts addressed student-driven topics. Students were not prompted to respond to any particular reading or lecture material. Four themes emerged from the discussion post codes, describing how veterinarians interact with the COVID-19 pandemic (Figure 2).

### Theme 1: Economic and Social Impacts of the Pandemic on Veterinarians

Fifteen students (63%) discussed the economic and social forces that impact the demand for veterinary services and the related effects of the pandemic. Four students (17%) predicted fewer pet owners would seek veterinary care because of decreased income secondary to the sudden surge of job layoffs or pandemic lock-downs (21). Two were concerned that pandemic and social disparities (22, 23) would result in some demographic groups struggling more than others to pay for their animals' veterinary care during the pandemic (Table 1). Although the demand for veterinary services sharply declined in April 2020 (24), it quickly rebounded and revenue increased compared to 2019 (25, 26). In a survey of emergency hospitals in March 2021, all responding hospitals reported a year-over-year increase in caseload, with many diverting non-life-threatening cases (27). Half of the hospitals planned to hire additional staff to address projected continued growth (27). Two students discussed that this increase might reflect pet owner opinions of veterinary care; as more owners view their pets as family members (28), veterinary services may be seen as essential rather than optional (Table 1). Students noted other pandemic-related social changes may increase veterinary services demand (Table 1). Six students (25%) observed that pet adoptions were increasing during stay-at-home orders and while more people were working from home, citing the mental and physical health benefits of the human-animal bond as contributing to the demand for pets. Although total adoptions actually decreased from March 2020 to December 2020 compared to 2019, the number of animal surrenders also decreased, resulting in an increase in the proportion of shelter animal live outcomes during the pandemic (29, 30). Students also noted that veterinary demand may increase because pet owners are more attentive to animal health problems when they spend more time with their pets. This is consistent with observed increased spending on pet products, calls to pet poison control hotlines, and client traffic to veterinary practices during the pandemic (31–33). Based on the students' observations, veterinarians may need to respond to fluctuations in revenue and demand, which may occur with natural disasters and economic cycles; training on business management and adaptation may be valuable to veterinary students.

**Table 1:** Representative quotes from discussion posts describing each theme.

**Theme**	**Representative Quotes**
Economic and social impacts of the pandemic on veterinarians	*Student 10: “Those who I am most concerned about, however, are those that were barely able to afford basic veterinary care before COVID-19 and have since reached an even more precarious financial state… I agree that most people view medical care for their animals as essential and that a decline of the veterinary profession as a result of this pandemic is unlikely”*
	*Student 16: “We must remember that, for pet owners, veterinary care is critical and thus can be considered a staple product, i.e. one that does not change in elasticity relative to economic outlook.”*
	*Student 14: “As this pandemic grows, shelters are seeing more and more adoptions and applications. Just recently, reports of a shelter in Colorado and … [the] SPCA of Erie County, NY…has emptied their dog kennels in response to people being home and wanting company”*
	*Student 4: “People are recognizing how living with animals is beneficial for their mental and physical health, both of which are direly in need of a boost in these uncertain times”*
	*Student 1: “During this period of social isolation, it makes sense that people seek to replace human companions with animal ones”*
	*Student 24: “With majority of clients staying home, they will probably be able to recognize issues with their pets sooner”*
Veterinarians adapted to challenges	*Student 12: “I think humans are creatures of convenience and post-pandemic there will be customer demand for continued use of telemedicine in veterinary and human medicine…I think there is a need for further legislation regarding the legalities of telemedicine and the AVMA stated that they support further regulatory efforts to help work this out.”*
	*Student 22: “[the] COVID-19 pandemic might end up being the catalyst that leads the veterinary field to accepting [telemedicine]… the current tele-medicine and decreased client interaction has the potential to decrease the trust between a client and their veterinarian.”*
	*Student 24: “there is a huge flaw with the ability to do [telemedicine] and that is misdiagnosing…A video call can easily distort what is actually occurring in person.”*
Opportunities for collaborations with veterinarians to advance public health	*Student 19: “Although veterinarians may witness cases of animal abuse that are linked to domestic abuse, they may not have much training in this field. Creevy et al. (34) proposed that partnerships between domestic violence shelters and veterinary school teaching hospitals can provide an educational platform for veterinary students, increasing their awareness on animal abuse and domestic violence.”*
	*Student 5: “Collaboration with veterinary researchers will help circumvent challenges already experienced with animal coronavirus and jumpstart the development of innovative solutions, based on shared mechanisms of these viruses.”*
	*Student 9: “Each of the posts in this thread have emphasized the importance of one health in combating disease outbreaks and I 100% agree that collaboration between the different groups is of utmost importance…the most important actions that veterinarians can take is to become more involved in policy and pressuring lawmakers to adopt the changes that science shows is necessary to safeguard us from future pandemics.”*
	*Student 8: “This outbreak, although it seems devastating in its impact, might actually be a good practice run for us as a scientific community of researchers, medical doctors, veterinarians, public health officials, epidemiologists, conservationists and more to come together and put [One Health] into action. Hopefully this outbreak will also foster greater collaboration between the scientific community, policy makers and the public.”*
	*Student 11: “[Future veterinarians] are at a unique position to use the attention brought to [wet markets] via COVID-19 to catalyze some real change, and if we can put enough pressure on the necessary government officials using both our scientific knowledge and our public health training, I believe we can make this happen.”*
Diverse perspectives are necessary for tackling pandemic-related disparities	*Student 10: “I also think we may see [sic] an even greater disparity in vet care between those above and below the poverty line…any health crisis disproportionately affects those with the lowest income”*
	*Student 11: “…many communities don't have another choice [other than eating wildlife], and we shouldn't marginalize those communities even more than they already are.”*
	*Student 1: “I would appreciate hearing potential ideas for how to end a deep-rooted cultural centerpiece like these wet markets without imposing Western cultural imperialism”*

Six veterinary students (25%) observed that large animal veterinarians have unique responsibilities and opportunities to address the pandemic's economic and social upheaval. For example, one student discussed the pandemic-related challenges in the dairy sector, noting that “production animal veterinarians have a very important role to play in supporting and guiding farmers” (Student 9). Another remarked that veterinarians' efforts to develop crisis operation plans, depopulation guides, and biosecurity protocols “highlights the veterinarian's role in one health as they are directly trying to maintain the health and welfare of animals that are crucial for the country's food supply” (Student 22). Overall, the students were optimistic about opportunities available for production-animal veterinarians to expand their roles by helping farmers overcome pandemic-related challenges. Students and veterinarians adapting to this new opportunity in production medicine may benefit from additional education on decision-making strategies, crisis management, and advocacy.

### Theme 2: Veterinarians Adapted to Challenges

Thirteen students (54%) discussed the benefits and challenges of new COVID-19 procedures in veterinary medicine, such as telemedicine, which was the most discussed adaptation (seven students, 29%). Students predicted that telemedicine would continue to be an important tool for small and large animal health after the pandemic (Table 1). The veterinary profession adapted rapidly over the last year to increase telemedicine capacity, and at least 30% of veterinary clinics surveyed by the American Veterinary Medical Association are using telemedicine to respond to pandemic related challenges (35). However, students identified persistent hurdles to the expansion of veterinary telemedicine, including the inability to diagnose many diseases without examining the animal, difficulty forming strong relationships with clients, and lack of legal and regulatory clarity (Table 1). While telemedicine is necessary and useful during the pandemic, the students' concerns indicate that further research is warranted to determine how telemedicine can be optimized to improve animal health and enhance veterinary medicine. The students also discussed the benefits and challenges of these new procedures, including the potential negative impact on veterinarians' well-being and mental health due to decreased social contact. This concern extended to the virtual learning adaptations for veterinary students; one second-year student noted that the stressful veterinary curriculum was being compounded by social isolation, fear, and anxiety. The veterinary profession has a higher suicide rate than the general population and, unfortunately, veterinary students are also reporting higher rates of depression, isolation, and anxiety during the pandemic (36, 37). Therefore, resources for well-being and resiliency are essential for veterinarians to adapt to the on-going challenges presented by the COVID-19 pandemic. For example, the AAVMC highlighted the importance of well-being considerations in university re-opening plans (38).

### Theme 3: Opportunities for Collaborations With Veterinarians to Advance Public Health

Eleven students (46%) remarked that veterinarians are key members of the global public health team and were hopeful that increasing collaborations with veterinarians could positively influence public health and safety, including social determinants of health (Table 1). They saw an opportunity for collaboration between veterinary researchers, laboratories, and pharmaceutical companies to develop SARS-CoV-2 diagnostic tests, therapeutics, and vaccines. Veterinarians have significant expertise in animal coronaviruses (39), which should be leveraged in the scientific response to the SARS-CoV-2 outbreak. Furthermore, veterinarians have been at the forefront of SARS-CoV-2 virology research (40, 41), epidemiology (5, 42, 43), diagnostic testing (4, 44), and vaccine development (45, 46). Ten of the students (42%) remarked that a team approach, with participants representing various areas of expertise, is necessary to address the ecological, biological, and social aspects of disease outbreaks. Four students extended their view of a veterinarian's role in public health to include political activism, social responsibility, and policy making (Table 1).

### Theme 4: Diverse Perspectives Are Necessary for Tackling Pandemic-Related Disparities

While the pandemic caused a cascade of complex problems, it is disproportionately affecting individuals from economically disadvantaged and non-Caucasian backgrounds (22, 23). As described in Theme 1, students recognized that these disparities play out in veterinary medicine as well (Table 1), which could result in increased stress, a decline in animal health, and result in some communities having fewer benefits from the human-animal bond. Two other students were concerned that policies to curb the COVID-19 pandemic and future outbreaks could have unequal impacts on different demographic groups. For example, in a discussion of regulating wet markets and wildlife consumption, they noted that policies to prevent disease spillover must be carefully crafted to avoid the marginalization of disadvantaged groups and cultural oppression (Table 1).

It will take a diverse group of people and perspectives to see and address these complex issues; however, veterinary medicine is one of the least diverse professions in terms of culture, ethnicity, and race in the United States (47) and does not reflect the diversity of animal owners (28). While the percentage of underrepresented minorities enrolling in veterinary school has increased from approximately 5% in 1980 to around 20% in 2020 (48), the veterinary profession still struggles to recruit and retain individuals from diverse ethnic, racial, cultural, and socioeconomic backgrounds. Lack of diversity and cultural competency could blunt the impact that the veterinary profession has on public health (49, 50). A recent call to address systemic racism in veterinary medicine specifically notes the need to improve diversity, equity, and inclusion in the profession for veterinarians to remain leaders in One Health (50). The CBVE framework recognizes the importance of diversity in veterinary medicine by making inclusivity and cultural competence a key component of veterinary education (51).

The alignment of student speculations with real-life trends may support the value of using such comments to inform professional change. For example, insights gained from this preliminary study may inform veterinary innovation, curriculum design, professional priorities, and business planning. Veterinary students represent a unique source of feedback and information as they are future leaders and representatives of the profession, are freshly educated with the latest clinical recommendations, and are unbiased by years of experience in the field. However, there are limitations of using information gathered from veterinary students, such as their lack of experiential knowledge. In the future, it would be valuable to evaluate the effectiveness and limitations of using student comments, in conjunction with a variety of other quantitative and qualitative sources of information, to create effective change in the profession. Caution is warranted in generalizing the results of this study due to the small sample size and limited representation of veterinary students (one cohort from one university). In particular, a larger percentage of students participating in this study were interested in careers outside of clinical practice than the U.S. veterinary student population, suggesting that non-clinical perspectives were overrepresented. In addition, a large proportion of participating students were interested in zoo, wildlife, and exotic animals. This could indicate self-selection bias; COVID-19 is a zoonotic disease spread by wildlife so those students may be more engaged and invested in the discussion. Future work should evaluate whether the themes identified in this cohort are in alignment with that of veterinary students across the country, with practitioners, and with industry partners.

## Conclusions

Our qualitative approach allowed us to identify four main themes from the second-year veterinary student discussion, which can be a starting point for additional investigations of how the profession can adapt, respond, and innovate during and after the pandemic. Although the veterinary students were concerned that the pandemic may negatively impact client finances and exacerbate existing disparities in veterinary medicine, they were generally optimistic about innovations to overcome pandemic-related challenges and opportunities for veterinarians to broaden the scope of their profession. Students noted that veterinarians could have a substantial impact on pandemic response and preparedness in collaboration with other scientific, medical, and policy experts. One of the limitations of this study is the small number of students who agreed to participate and localization of participants to one veterinary institution. The small sample size and limited study population reduces the diversity of opinions and perspectives represented in our themes, although the topics covered were generally representative of the overall class discussion and we achieved code saturation. Student responses were in alignment with real trends that were emerging during the early pandemic. Thematic analysis of the responses identified many topics that may benefit from further exploration so that veterinarians can prepare for a rapidly changing professional landscape.

## Data Availability Statement

The raw data supporting the conclusions of this article will be made available by the authors, without undue reservation.

## Ethics Statement

The studies involving human participants were reviewed and approved by Cornell University Institutional Review Board (protocol ID# 2005009621). The patients/participants provided their written informed consent to participate in this study.

## Author Contributions

CL, AH-B, and CC contributed to the conceptualization and design of the study. CC collected and organized the data. CC and CL analyzed the data. CL wrote the original draft. All authors contributed to the interpretation of the results, manuscript revision, and approved the submitted version.

## Funding

Publication of this work was supported by the Cornell University Graduate School Future Faculty and Academic Careers office.

## Conflict of Interest

The authors declare that the research was conducted in the absence of any commercial or financial relationships that could be construed as a potential conflict of interest.

## Publisher's Note

All claims expressed in this article are solely those of the authors and do not necessarily represent those of their affiliated organizations, or those of the publisher, the editors and the reviewers. Any product that may be evaluated in this article, or claim that may be made by its manufacturer, is not guaranteed or endorsed by the publisher.
