# Supplementary material for: Brief Research Report: Veterinary Student Perspective on COVID-19 and Veterinary Medicine
Source: Front Vet Sci. 2021 Oct 14;8:723890. doi: 10.3389/fvets.2021.723890 (PMC8551393; doi:10.3389/fvets.2021.723890)
Supplement: Supplementary file 2 [file Table_2.DOCX]

**Abbreviated Syllabus**

Course 5705/VIIIe

PUBLIC HEALTH

Spring 2020: March 30, 2020 – May 15, 2020

**COURSE OBJECTIVES**

A major goal of the course is to highlight the DVM’s public health role. Our training and expertise places us at the interface between humans and animals, making us advocates for both. Our professional oath reminds us of this duality with inclusion of the phrase, “for the benefit of society.” Lecture topics have been chosen to provide a broad overview of public health activities and opportunities.

**COURSE COMPONENTS**

There are five components to this course:

1. Lectures on selected topics and complementary readings
2. Public health newsletters
3. Initial Accreditation Training
4. Zoonotic diseases independent study
5. Online discussion of COVID-19: “What Veterinarians Need to Know”
6. **Lectures on Selected Topics**:

| **Date** | **Topic** |
| --- | --- |
| 3/31 | Large Animal Practice and Public Health |
| 4/2 | Neglected Zoonotic Diseases |
| 4/2 | Environmental Impacts of Animal Agriculture |
| 4/3 | Packing and Shipping Biologic Specimens |
| 4/3 | Pet Food: Recalls and Product Safety |
| 4/15 | Rabies |
| 4/16 | Dog Bites/Zoonoses |
| 4/17 | Microbial Safety of Animal-Based Foods |

1. **Public Health Newsletters**

Students are expected to sign up to at least two newsletters during the Public Health course. You are encouraged to maintain and expand these subscriptions to stay up to date on relevant professional information. You will submit the subscription confirmation emails to the Canvas assignment.

1. Worms and Germs blog: <https://www.wormsandgermsblog.com/>
   1. Subscribe in the yellow right-hand column
2. ProMED mail: <https://isid.org/promedmail-subscribe/>
   1. Subscribe to ProMED or ProMED-digest (first two options)
3. CDC newsletters: <https://tools.cdc.gov/campaignproxyservice/subscriptions.aspx>
   1. Many are available. Some of veterinary interest include: Antibiotic Resistance & Antibiotic Use; Animal Importation Regulations; Food Safety; Public Health Law News; One Health; Healthy Pets, Healthy People
4. **Veterinary Accreditation**

All students are responsible for completing the Initial Accreditation Training (IAT) and emerging/exotic disease online course. This training is the first step in becoming a USDA Accredited Veterinarian.

1. **Zoonotic Diseases of Importance in Veterinary Medicine**

There are four independent study resources:

1. “Compendium of Veterinary Standard Precautions for Zoonotic Disease Prevention in Veterinary Personnel”
2. “Zoonotic Diseases of Importance in the U.S.” PowerPoint
3. Technical Fact Sheets for selected diseases from The Center for Food Security & Public Health
4. “Compendium of Animal Rabies Prevention and Control, 2016”
5. **Online Discussion of COVID-19**

As we are learning about public health, coincidentally we are in the middle of a public health emergency caused by the virus SARS-CoV-2. Although this virus appears to affect only humans, it will also impact veterinarians in many ways: zoonosis questions from clients, shortages of supplies and drugs, ill clients and staff, economic impacts of social distancing, and disrupted travel, to name a few. It is important that veterinarians inform themselves about the COVID-19 outbreak and are prepared to answer questions or participate in the public health response as needed.

Students are expected to actively participate in this discussion. Discussion prompts will be provided but students may add their own topics as long as they are relevant to veterinary medicine. **At a minimum, students must provide 2 substantive posts, at least 1 of the posts must be a response to another student. See the course website for a description of a substantive post and grading rubric.** If you post more than twice, your two highest scoring posts will be used.

**GRADING:**

Public Health Newsletter Subscriptions: 10%

Initial Accreditation Training and Disease Modules Completed: 15%

Zoonotic Diseases Closed-Book Pre-Test: 5%

Zoonotic Diseases Open-Book Quiz: 20%

COVID-19 Discussion Participation: 25%

Lecture and Reading Material Open-Book Quiz: 25%

**Discussion prompts provided by the course instructor and student-driven discussion topics.** The seven underlined prompts were addressed by at least one of the discussion posts included in this study.

**Initial discussion prompt from instructor:**

*“As we are learning about public health, coincidentally we are in the middle of a public health emergency caused by the virus SARS-CoV-2. Although this virus appears to affect only humans, it will also impact veterinarians in many ways: zoonosis questions from clients, shortages of supplies and drugs, ill clients and staff, economic impacts of social distancing, disrupted travel, to name a few. It is important that veterinarians inform themselves about the COVID-19 outbreak and are prepared to answer questions or participate in the public health response as needed…”*

**Potential discussion topics provided by the instructor throughout the course:**

- Reducing risk of zoonoses
- COVID-19 testing in pets
- COVID-19 infections in pets
- Anthropozoonotic spread of COVID-19 to pets
- Canine coronavirus vaccine
- Rabies clinics during the pandemic
- Drug and supply shortages
- Shutdown of meat processing plants
- Impacts on veterinary research
- Homemade PPE
- Vet clinic “re-opening”
- Upper respiratory disease in animals at risk of COVID-19

**Student-driven discussion topics:**

- Wet markets and cultural differences
- Pet adoption
- Virtual learning
- COVID-19 infections in wildlife, zoo animals, and exotic animals
- Other coronavirus infections in animals
- Horse racing and lockdowns
- Production animal market disruptions, impact on farms and consumers
- Emergency response and veterinarians
- Essential vs elective veterinary care
- Antimicrobial stewardship
- Euthanasia
- Telemedicine
- Evidence-based medicine
- Animal cruelty and domestic abuse
- Environmental and wildlife conservation
- Mental health and wellness

**COREQ Checklist for: *Veterinary Student Perspective on COVID-19 and Veterinary Medicine***

Candice B. Limper, MS (ORCiD: 0000-0001-6945-069X), Ariana Hinckley-Boltax, DVM (ORCiD: 0000-0002-2867-9233), and Casey L. Cazer, DVM PhD (ORCiD: 0000-0002-2290-1868)

| **Domain 1: Research Team and reflexivity** |  |
| --- | --- |
| **Personal Characteristics** |  |
| 1. Interviewer/facilitator | Casey Cazer supervised the online course discussion |
| 1. Credentials | Casey Cazer, DVM, PhD |
| 1. Occupation | Veterinary Epidemiologist, Course Leader |
| 1. Gender | Female |
| 1. Experience and training | Training in evidence-based instruction; PhD in epidemiology |
| **Relationship with participants** |  |
| 1. Relationship established | Relationship was established prior to study commencement |
| 1. Participant knowledge of interviewer | Participants knew the facilitator as the course instructor but the research began after the course concluded |
| 1. Interviewer characteristics | Facilitator was interested in this topic because of the massive social and economic changes happening during the online course |
|  |  |
| **Domain 2: Study Design** |  |
| **Theoretical Framework** |  |
| 1. Methodological orientation, theory | Content and thematic analysis |
| **Participant selection** |  |
| 1. Sampling | Convenience |
| 1. Method of approach | Email |
| 1. Sample size | 24 |
| 1. Non-participation | 89 students did not respond to email recruitment, reasons unknown |
| **Setting** |  |
| 1. Setting of data collection | University |
| 1. Presence of non-participants | Non-participants took part in the course discussion |
| 1. Description of sample | Demographic data not available; discussion occurred online using learning management system from March 30, 2020 to May 15, 2020 |
| 1. Interview guide | Some discussion prompts provided (see manuscript) |
| 1. Repeat interviews | No |
| 1. Audio/visual recording | No |
| 1. Field notes | No |
| 1. Duration | Discussion posts averaged 454 words |
| 1. Data saturation | Yes – code saturation was achieved when each coder analyzed posts from 10 random students. No additional codes were needed in the full analysis. |
| 1. Transcripts returned | No – posts were written by students |
|  |  |
| **Domain 3: Analysis and Findings** |  |
| **Data Analysis** |  |
| 1. Number of data coders | 2 |
| 1. Description of the coding tree | See supplementary data code book |
| 1. Derivation of themes | Derived from data |
| 1. Software | ATLAS.ti (version 8, Scientific Software Development GmbH, Berlin, Germany) |
| 1. Participant checking | No |
| **Reporting** |  |
| 1. Quotations presented | Yes, with participant number |
| 1. Data and findings consistent | Yes |
| 1. Clarity of major themes | Yes |
| 1. Clarity of minor themes | N/A |
